# Evaluation of the efficacy of rotary and reciprocating systems for the removal of filling material for various root canal filling techniques

**DOI:** 10.34172/joddd.2023.36695

**Published:** 2023-07-17

**Authors:** Neslihan Büşra Keskin, Selen İnce Yusufoğlu

**Affiliations:** Ankara Yıldırım Beyazıt University, Faculty of Dentistry, Department of Endodontics, Ankara, Turkey

**Keywords:** Retreatment, Single cone, Gutta core, Warm vertical condensation, Reciproc

## Abstract

**Background.:**

This study aimed to evaluate the efficacy of various filling and retreatment techniques for oval-shaped root canals.

**Methods.:**

Sixty distal roots of mandibular molar teeth were included in the study. The roots were prepared using the ProTaper Next (PTN) X4 rotary system and irrigation with 2.5% NaOCl. The roots were then divided into three groups and filled with Total Fill BC Sealer (FKG Dentaire) using three different techniques (n=20): single cone (SC), GuttaCore (GC), and warm vertical condensation (WVC). The retreatment procedure was performed using two different instrumentation techniques: Reciproc 50 (R50) and PTN X5 (n=10). To analyze the remaining filling material, the roots were divided buccolingually in two parts with the help of diamond separators, and photographs were taken at x16 magnification using a dental operating microscope (DOM). The ratio of the remaining filling materials was calculated using image analysis software and statistically evaluated using the Kruskal–Wallis test.

**Results.:**

None of the assessed retreatment procedures completely removed the filling materials from the root canals. For both instrumentation techniques, more root canal filling material remained in the WVC group (*P*<0.05).

**Conclusion.:**

The GC filling technique had a higher cleaning percentage than the WVC and SC techniques in the coronal region. The R50 system was found to be superior to the PTN X5 system for retreatment, and the root canal fillings applied using the WVC technique were more difficult to remove than the fillings applied using the other techniques.

## Introduction

 Orthograde retreatment is the primary target of unsuccessful root canal treatments. In the retreatment procedure, firstly we aimed to remove contaminated root canal filling materials and then, the chemomechanical instrumentation of the root canal is repeated, and the refilling processes are performed properly.^1^ Various endodontic systems and techniques, such as hand files, Gates Glidden burs and conventional and reciprocal rotary systems, have been described for the removal of root canal fillings.^2,3^ However, none of these methods have been found to completely remove root canal filling.^2,3^ This is particularly difficult in teeth with complex root canal anatomy and an oval shaped root canals.^4^

 Many filling techniques can be used for endodontic treatment; however, the most commonly used technique is the lateral condensation technique.^5,6^ Although it is easier to check the working length while using the lateral condensation technique, it cannot obturate root canal irregularities better than warm root-filling techniques.^6^ Warm obturation techniques have been advanced to provide better adaptation of the filling materials to the root canal by obtaining three-dimensional obturation, with the advantage of lower cavity formation compared to cold compaction techniques.^7^ The warm vertical condensation (WVC) technique, having a lower void percentage compared to the cold lateral condensation technique, provides a more homogeneous root canal filling.^8^

 It has been reported that the GuttaCore (GC) system (Dentsply Sirona, Switzerland), a core-carried system, can be easily removed using rotary instruments because of its low modulus of elasticity, making it easy to break under torsional forces.^9^

 Kinematic movements can affect the retreatment procedures. Several studies have shown that reciprocal movement is more effective than conventional movement for retreatment procedures.^10,11^ The Reciproc (R) system (VDW, Münich, Germany) is recommended by the manufacturer for the retreatment cases due to its reciprocal movement and flexible S-shaped cross section. Current study aimed to investigate the removal performance of ProTaper Next (PTN) and R systems which root canals obturated with using single cone (SC), WVC, and GC filling techniques and under dental operatiing microscope (DOM). The first null hypothesis of this study is that there will be no difference between the two instrumentation systems in terms of removing root canal filling materials. The second null hypothesis is that there will be no difference between the remaining filling materials according to the different filling techniques.

## Material and Methods

 Sixty distal roots of mandibular molar teeth extracted for orthodontic or periodontal reasons were included in the study. Radiographs were taken of the teeth. Only oval-shaped root canals were included in the study; additional teeth were added to replace those that were not included in the study. The roots were examined under x10 magnification with a DOM (Leica M3: Ernst Leitz GmbH, Germany) to identify any cracks, resorption, or calcification. The samples were kept in distilled water at 4°C until required.

###  Root canal preparation

 After preparing access cavities for the teeth, a #10K file (Dentsply Sirona, Switzerland) was placed in the root canal and advanced until it was visible from the apical. The working length was determined. All the root canals were prepared with PTN instruments up to X4 (Dentsply Sirona) according to the manufacturer’s instructions (torque setting = 2.5 Ncm and speed setting = 300 rpm) using an endodontic motor (SybronEndo; Orange, CA, US). 2 ml 2.5% NaOCl (Werax; Izmir, Turkey) was used for irrigation after each filling. For the final irrigation, 2 mL 17% EDTA (Werax) and 2 mL distilled water were used, and the root canals were then dried with paper points (Diadent, DiadentGroup International, Burbany, BC, Canada). For the root canal filling, the teeth were then randomly separated into three groups (n = 20).

###  Root canal obturation

*Group 1:* Single cone (SC) technique: Apical size 40 Total Fill BC master cones (FKG Dentaire, Switzerland) were tugged back. The master apical file as covered with Total Fill BC root canal sealer (FKG Dentaire), and the root canals were obturated according to the SC.

*Group 2: *GuttaCore (GC) technique: Total Fill BC Sealer (FKG Dentaire) was placed on the root canal walls using paper points. GuttaCore 40.04 (Dentsply Sirona) was used by adjusting it to the working length. According to the manufacturer’s recommendations, the gutta percha was heated in an oven (ThermaPrep 2; Dentsply Sirona) to soften it before being inserted in the root canals 0.5 mm shorter than the working length. A round diamond bur was used to carve off the system’s handle component.

*Group 3: *WVC technique: Suitable plugs that can penetrate the root canal at a length shorter than 0.5 mm of the working length were used. The apical size 40 Total Fill BC master cones were tugged back. Following that, The Total Fill BC root canal sealer was applied to the gutta-percha (45.04; FKG Dentaire), which was then advanced till it reached the working length. With a heated plugger (Calamus Dual 3D Obturation System; Dentsply Sirona) set to 200°C, the root canal fillings were removed, leaving 5 mm of gutta percha in the apical region. Carrying gutta percha (Calamus Double 3D Obturation System) was used to obturate the other parts of the root canal fillings, and the heated plugger was used to compress the gutta-percha in the apical direction.

 For all the samples, temporary filling material (Cavit G; 3M ESPE, Seefeld, Germany) was used for closing the cavities. The samples were stored at 37°C and 100% humidity for one week to ensure that the root canal sealer was completely setting. The samples were then divided into two subgroups for the retreatment procedure (n = 10). The retreatment procedure was performed in two groups using root canal files X5 and R50.

1. *X5 group:* The X5 instrument was used with a crown-down technique. The procedure was performed by applying slight pressure toward the apical until the root canal filling of the working length was cleared.
2. *R50 group:* The R50 instrument was used in a reciprocating motion until the working length was reached. After three pecking movements, the file was removed from the root canal and irrigated. This procedure was performed until the root canal filling of the working length was cleared.

Irrigation was performed between each instrument using 2.5 mL 2.5% NaOCl, 2.5 mL 17% EDTA and 2.5 mL distilled water were used for the final irrigation. The same irrigation protocol was used for all the groups.

###  Microscopic evaluation

 The roots were divided buccolingually into two parts with a diamond separator, and the ratio of remaining materials were determined using the DOM at × 16 magnification. The photos were uploaded to a computer, and image analyses software was used to calculate the amount of residual filling material (mm^2^) (ImageJ, Version 7; Wayne Rasband, NIH, MD, US).

###  Scanning electron microscopy analysis (SEM)

 Each group had two randomly selected samples processed for SEM (Quanta FEG 400; FEI Co., US) analysis. The root halves were attached to blades made of aluminum (Silverpaint; Agar Scientific Ltd., Stansted, Essex, UK) and then they were dried out using ethanol solutions. The samples were coated and examined under × 2000 magnification.

###  Statistical analysis

 The data were analyzed using SPSS, version 22 (SPSS Inc., Chicago, IL). The normality of the variables was tested using the Shapiro–Wilk test. Because the data were non-parametric, the evaluation between the groups was undertaken using Kruskal–Wallis and binary comparisons using the Mann–Whitney U test (*P* < 0.001). The level of statistical significance was set at 5% (*P* < 0.05).

## Results

**Figure 1:**
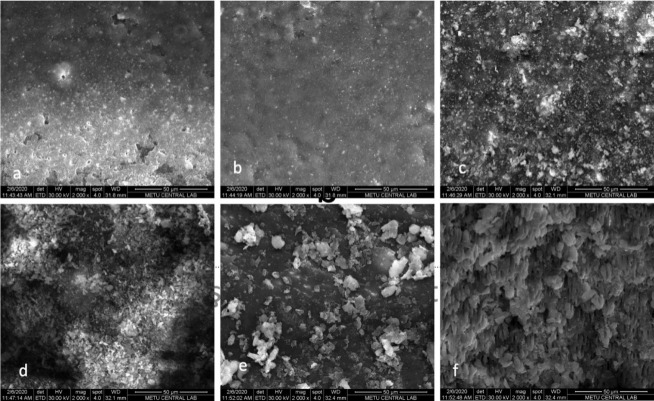
Images obtained by SEM at × 2000 magnification a) SC-R50, (b) SC-F5, (c) GC-R50 (d) GC-F5, (e) WVC-R50, (f) WVC-F5

 The means and standard deviations for all the groups are presented in Table 1. Residual filling material was found in all groups. There were statistically significant differences between obturation techniques (*P* = 0.07). There were statistically differences between WVC and GC obturation techniques. WVC X5 = WVC R50 < GC X5 (*P* = 0.001, *P* = 0.011) and WVC R50 < GC R50 (*P* = 0.01). SC R50 = SC X5 > WVC R50 (*P* = 0.004, *P* = 0.017). SC R50 = GC X5 > WVC X5 (*P* = 0.046, *P* = 0.011). Although there was no statistically significant difference between the root canal files, it was observed that the most residual filling material was in the WVC group in both file types (Figure 1). There was no statistical difference between the other experimental groups (*P* > 0.05).

**Table 1:** Mean and standart deviations of all groups

	**R50**	**F5**
SC	82 ± 3.41^a^	80.7 ± 5.12^a^
GC	80.5 ± 3.93^a^	86.9 ± 2.85^a^
WVC	60,9 ± 5.49^b^	66.9 ± 6.14^b^

SC: Single cone; GC: GuttaCore; WVC: Warm vertical condensation Values with different superscript letters in each column were statistically different at *P*= 0.05.

## Discussion

 The existence of old filling material negatively affects the success of retreatment therapy. It is important for clinicians to choose the fastest and most effective technique for retreatment procedures. In this study, two different instrumentation systems and different obturation techniques were used to assess the amount of remaining root canal filling material after retreatment procedure.

 Previous researches showed that clinicians cannot entirely remove gutta percha/sealer from root canals regardless of instrumentation techniques.^12-14^ Similarly, the instrumentation techniques used in the current study could not completely remove the filling materials from the root canals. Some studies have reported that rotary and reciprocating systems remove more filling material, while other studies have found either rotary files^7^ or reciprocating systems to be superior.^10,15^ Some studies have observed that rotary and resiproc file systems remove filling material in a similar manner.^4^ However, in the current study, X5 and R50 file systems were used for the retreatment. The R50 was superior to the X5; therefore, the first hypothesis was rejected. We thought that the use of reciproc files with S-shaped cross sections and reciprocating motion might be more effective for further removing root canal filling material. The differences in the outcomes of these previous studies could have been the result of many influencing factors, such as the application of different obturation techniques, different production and cross-sectional characteristics of the instrumentation systems, differences in the morphological properties of the applied root canals, and the operator’s experience.

 In our study, the root canal preparation was completed using a PTN X4 file, and the apical diameters of the samples were 40.04. Size 50.04 files were used for the retreatment procedures and to remove the residual filling materials remaining in the dentin tubules. A previous study found that the additional preparation of root canals using larger file systems did not remove the filling any better.^16,17^ The current study supported this outcome: although larger sized files were used, the root canal filling material was not entirely removed from all the samples.

 In order to transfer the well-documented bioactivity and biocompatibility of di and tri calcium silicate cements to root canal sealers, endodontic sealers incorporating calcium silicate formulations have recently been developed. Total Fill BC Sealer is a monophasic root canal sealer and contains monobasic calcium phosphate and tricalcium silicate. It needs an external fluid, like tissue fluid, to harden.^18^ It has been reported to have good sealing properties and the ability to release calcium ions.^19^ In the current study, Total Fill BCroot canal sealer was used because of its ideal and maximum sealing properties.

 There are no studies in the literature that investigate remaining filling material after retreatment procedures of root canals obturated using different obturation techniques. According to the current study’s results, the root canals filled using the WVC technique regardless of the file type showed more residual filling material in all parts of the root canals after the retreatment procedure compared with the other obturation techniques. For this reason, the study’s second null hypothesis was rejected. A previous study reported that although the WVC technique created tighter filling than SC and lateral condensation techniques, there was no statistically significant difference.^20^ Alim and Garip Berker^21^ stated that the lateral condensation technique showed superior filling in apical and middle sections compared to the SC and WVC techniques. Evaluating the different obturation techniques using micro computed tomography analysis (micro-CT), they found no statistically significant difference between the filling techniques in the apical, middle, and coronal regions.

 Previous studies have found the SC technique to be less successful in terms of gap than other filling techniques^21,22^; however, in the current study, there was less remaining root canal filling material in the SC group. Standard round gutta percha cones can be removed more easily from the root canal due to a lack of full compatibility with canal walls. Previous studies have stated that there is less microbial leakage and more successful clogging of the lateral canals using continuous heat obturation techniques.^23^ According to Alim and Garip Berker,^21^ the GC technique has a better filling feature than the WVC technique. In the current study, the GC technique resulted in more cleaned areas in the coronal region, and because of using a core-bearing material, we can also attribute lower GC adaptation to the coronal part.

 Remaining filling materials can be evaluated using various imaging techniques, such as X-ray, stereomicroscopy, a DOM, CBCT, and micro-CT.^13,14^ The remaining root canal filling material was assessed using a DOM in the current investigation. Although DOM images produce two-dimensional enlarged images, they are insufficient for evaluating the amount of remaining filling material in dentinal tubules. This was the limitation of the current study.

## Conclusion

 According to the study’s results, there were no completely clean root canals after the retreatment procedures. While the R 50 file system was more effective in removing root canal filling material than the X5 system, it was observed that the root canals filled using the WVC obturation technique resulted in more residual filling materials than the other obturation techniques regardless of the instrumentation systems.

## Competing Interests

 No competing interests.

## Ethical Approval

 This study design was approved by the ethics committee of Ankara Yıldırım Beyazıt University (No: 2020-172).

## Funding

 No funding.
